# The role of home medication storage location in increasing medication adherence for middle-aged and older adults

**DOI:** 10.3389/fdgth.2022.999981

**Published:** 2022-11-04

**Authors:** Lisa Gualtieri, Eden Shaveet, Brandon Estime, Avi Patel

**Affiliations:** Department of Public Health and Community Medicine, Tufts University School of Medicine, Boston, MA, United States

**Keywords:** medication adherence, older adults, digital health, medication management, medication storage, adherence devices

## Abstract

**Background:**

Over 50% of US adults do not take their prescriptions as prescribed, which is responsible for 33%–69% of hospital admissions and 125,000 deaths annually. Given the higher prevalence of prescription drug use among middle-aged and older adult populations, promoting medication adherence is of particular importance with these age groups. Two speculated facilitators of medication adherence are home medication storage location and the use of digital health devices.

**Objective:**

Our objective was to use survey data to investigate the associations between medication storage location and medication adherence among adults 40 years and older. Additionally, we aimed to report preliminary findings about the associations between use of devices and medication adherence in this same population.

**Methods:**

We conducted primary analysis of data sampled from a home medication management survey deployed in November 2021 (*n* = 580). We conducted exploratory analyses by way of chi^2^ tests and creation of bivariate logistic regression models.

**Results:**

The most commonly used storage locations by our sample were nightstand drawers (27%), kitchen cabinets (25%), and atop bedroom nightstands (23%). Several medication storage locations were significantly associated with decreased odds of having ever forgotten to take a medication, including kitchen drawers, in refrigerators, atop bedroom nightstands, in nightstand drawers, and backpacks, purses, or bags. Two home medication storage locations were significantly associated with increased odds of having ever forgotten to take a medication: kitchen cabinets and bathroom vanities. Further, most (94%) survey respondents indicated they would be receptive to guidance about where to store their medications.

**Conclusions:**

Given that some home medication storage locations are associated with adherence, an intervention to guide storage location selection may support increased adherence, especially with high receptivity expressed for such guidance. Increased adherence may also accrue from device usage paired with optimized home medication storage location. We plan to investigate that further, as well as how new device designs can incorporate contextual cues related to location to promote medication adherence more effectively in middle aged and older adults.

## Introduction

### Definition and importance

Medication adherence, the extent to which a patient follows a stipulatory medication treatment plan, is crucial to the success of patient care and is indispensable in reaching clinical goals (1). However, only 50% of people adhere to medication guidelines (2), posing substantial risk to patient health and safety. Medication nonadherence is responsible for as many as 33%–69% of hospital admissions and 125,000 deaths annually (3). Other consequences of medication nonadherence include waste of medication, disease progression, and a lower quality of life overall (4). There is a rising trend in medication nonadherence across all age, sex, and racial groups in the United States (5), which is particularly concerning for middle-aged and older adults as the likelihood to be prescribed long term medications increases with age (6, 7).

Medication nonadherence, while well-studied, continues to be a vexing issue for clinicians and researchers (2, 8). In 2003, the World Health Organization (WHO) indicated that improved medication adherence interventions may have greater impact on population health than specific medical treatments (9). Known barriers to medication adherence include out-of-pocket costs of medications, physical difficulties obtaining a medication supply, and difficulty following intake guidance (10, 11). Patients facing multiple, co-occurring barriers are even less likely to adhere to prescribed medications (12).

### Home medication management

Given the importance of contextual cues and mental associations in developing routines (13), medication storage locations may be a vital component of improving medication adherence across populations. In the process of habit formation, settings associated with certain behaviors may influence an individual's actions to deviate from conscious motivation (14). External contextual cues in a person's environment trigger an automatic response that promotes habit formation (15). Previous work exploring strategies for medication adherence found that reliance on multiple cues may have a beneficial impact on adherence to medication regimens (16). Locations where medications are frequently stored may have the capacity for a plethora of contextual cues.

Understanding a patient's home medication management routine, including home medication storage location selection, may be needed for more effective medication management and strategic placement of devices that serve to dispense or remind to take medications. For example, devices that rely on auditory or visual cues need to be in locations where patients can hear or see the alerts and notifications. While home medication management is well-studied in relation to patient safety (17), less is known about the role of home medication storage location in medication adherence.

### Patients and locations of interest

Behavior change theories such as the Rubicon model of action phases suggest that the level of intent in improving medication adherence should be considered when developing medication adherence interventions (18). According to this model, interventions are most effective when an individual intends to be adherent to their medication but only needs additional support and guidance to do so (19). As a result of exploring this theory, our primary interest is in patients who do not face well-documented barriers to medication adherence, such as cost and access (11), and intend to be adherent, yet still struggle to achieve adherence (19).

Our population of interest is middle-aged and older adults as this demographic is more likely to take one or more prescription medications compared to other age groups (20). For some health conditions, such as hypertension and diabetes mellitus, a patient's older age may also impact their level of adherence (21).

Our location of interest is in patients’ homes as more medications are taken at home than in hospitals and clinics combined (1, 22). Our research focuses on understanding the barriers to medication adherence in the home to design aids that increase adherent behavior. We explore this by first understanding where patients store their medications, and then by investigating how patients select these locations. We then investigate whether the locations themselves or the determinants of selection correlate with adherence. Finally, we examine factors related to home medication management including the use of digital aids to help with adherence.

### Home medication storage locations

Prior research into home medication storage has examined location with respect to safety, climatic conditions, and routines (23–25); however, the impact of home storage locations on medication adherence is understudied in literature. A study of medication storage conditions found that, of 170 participants aged 65 and older, 76% complied with drug product label recommendations for temperature, light, and humidity (26). A study on medication disposal found that 81.5% of respondents in 445 telephone interviews had prescription medications and almost all the respondents indicated that there were excess and leftover medications in their homes (23). Another study on safe medication storage surveyed 1,074 people aged 50–80 with grandchildren aged 0–17 (27). Their findings indicated that 89% of respondents had prescription medications in their homes and 84% reported that they kept their medications in the same place they typically store them when their grandchildren visited (27).

MedlinePlus, an online information service produced by the United States National Library of Medicine, suggests storing medications in the dresser drawer, kitchen cabinet, storage box, shelf, or closet (28). Although they do not reference medication storage location relative to medication adherence, they state that location plays a role in medication effectiveness and safety. With 418 million users in 2021 (29), MedlinePlus, and services like it, could influence medication storage decisions.

A few studies have addressed the role of strategies, routines, and habit formation in establishing a successful medication regimen (16, 30). One study found that older adults employ both internal (e.g., use of mental associations) and external (e.g., use of physical objects and/or locations) strategies to remember to take their medications (16). Another study considered the use of contextual cues as an aid to develop a new behavior, reporting that patients who store their medications in locations that are conducive to their routines were more likely to be adherent to their medication regimens (13). Behavioral interventions that provided counseling on adherence strategies considered the storage of medications only as a cue to remind individuals to maintain adherence (31, 32), yet their effect was modest. Survey respondents who used visible cues throughout the day were unable to remember whether they had taken their medication (13).

Several efforts have been dedicated to the development and use of devices to improve medication adherence, including designing digital health devices and apps to dispense medication and/or remind users that it is time to take medication (13). However, simple, low-cost devices have yet to produce clinically impactful outcomes (33). There is an increasing number of devices aimed at improving adherence, yet sub-optimal placement of these devices in the home may lead to less efficacious changes in adherence.

No study has evaluated the relationship between medication storage locations and adherence nor the relationship between storage location and device use. Given these gaps in the literature, we designed and deployed a survey to learn more about medication management in the home. Namely, we aimed to assess whether there exists a bivariate relationship between home medication storage location and self-reported medication adherence. Additionally, we aimed to assess the bivariate relationships between use of digital devices for medication intake reminders and self-reported medication adherence. Our first hypothesis is that middle-aged and older adults store their medications in multiple locations while our second hypothesis is there is an association between home medication storage locations and self-reported medication adherence.

## Methods

### Study sample

We collected data *via* deployment of the Home Medication Management Survey, designed by members of the Digital Health Research Group at Tufts University, and fielded between November 18 and December 14, 2021. We deployed the survey in English *via* Google Forms, an online, cloud-native survey-development platform that encrypts files in transit and at rest (34). We recruited participants *via* informational posts on social media platforms, including Twitter, Facebook, Instagram, and LinkedIn, and *via* the Osher Lifelong Learning Institute at Tufts University electronic mailing list, which reaches an audience of around 2,000 older adults, primarily in the Greater Boston area. Eligible participants for the survey were 18 years of age or older with access to an internet-enabled device. Upon completion of the survey, participants could elect to enter a drawing for one of five Amazon gift cards valued at $25. All study protocols were reviewed and approved by the Tufts University Health Sciences Institutional Review Board in Boston, MA.

### Procedures

We recorded a total of 1,966 survey responses at the close of the survey and deemed 1,673 (85%) of responses to be valid after excluding responses on suspect of fraudulence. Dropped responses met any of the following exclusion criteria: responses that were not in English (49 responses dropped); consecutive sets of identical responses posted at the same time (132 responses dropped); responses containing an abnormal email address for the drawing that included a long string of numbers we suspected to be fraudulently generated (19 responses dropped); or responses that included suspicious identical open-text responses within the same day (83 cases dropped).

Of the 1,673 remaining eligible responses, our study sample for this paper was middle-aged and older adults meaning respondents indicating an age of 40 years or older (*n* = 580). We selected this age range to adhere with the centers for disease control and prevention's inclusion of 40–79 years in their report of prescription drug use (35). However, we did not exclude respondents 80 years of age and older for sample size considerations.

### Measures

We designed survey questions to learn about respondents’ experiences with medication management in the home. Questions assessed use of aids to adherence, perceived importance of adherence, self-reported adherence, as well as demographic items.

#### Demographic variables

Demographic items included in our analysis were age in years by decade (40–49, 50–59, 60–69, 70–79, 80–89, 90+), race and ethnicity (multi-select categorical item), sex, and highest level of education completed. Table 1 displays the demographic distribution of our sample.

**Table 1:** Sample characteristics by self-reported adherence (*n* = 580).

	Overall N (%)	Ever forgot to take a medication (row %)				Recently forgot to take a medication^a^ (row %)			
Characteristic		Yes	No	*chi^2^* *	*p*	Yes	No	*chi* ^2^ *	*p*
Overall	580 (100)	72	28			35	65		
Age by decade									
40–49	244 (42)	59	41	38.76	<0.001	39	61	8.36	0.079
50–59	127 (22)	75	25			35	65		
60–69	82 (14)	85	15			37	63		
70–79	84 (14)	86	14			23	77		
≥ 80^b^	43 (7)	81	19			28	72		
Race/Ethnicity^c^									
White	439 (76)	75	25	10.20	0.001	32	68	5.37	0.02
American Indian or Alaskan Native	70 (12)	49	51	20.65	<0.001	36	64	0.05	0.817
Black or African American	41 (7)	63	37	1.44	0.231	49	51	3.99	0.046
Asian	27 (5)	85	15	2.59	0.108	59	41	7.69	0.006
Hispanic or Latino	27 (5)	74	26	0.09	0.766	44	56	1.24	0.265
Native Hawaiian or Other Pacific Islander	12 (2)	75	25	0.07	0.789	67	33	5.62	0.018
Sex^d^									
Female	345 (59)	77	23	12.87	0.002	34	66	3.96	0.138
Male	233 (40)	64	36			35	65		
Highest Education Completed									
<High school diploma or equivalency	17 (3)	82	18	34.72	<0.001	59	41	12.30	0.056
High school diploma or equivalency	45 (8)	53	47			38	62		
Some college	82 (14)	51	49			32	68		
Associate's degree	47 (8)	68	32			49	51		
Bachelor's degree	160 (28)	74	26			29	71		
Master's or professional degree^b^	183 (32)	80	20			33	67		
Doctoral degree	46 (8)	83	17			39	61		
Digital Reminder Methods Used^c^									
Any digital method^e^	325 (56)	60	40	48.46	<0.001	36	64	0.48	0.489
Smartphone app or alarm^b^	224 (39)	63	37	14.69	<0.001	37	63	1.07	0.301
Apple Siri	86 (15)	45	55	34.06	<0.001	35	65	0.01	0.932
Amazon Alexa	42 (7)	60	40	3.22	0.073	50	50	4.83	0.028
Smartwatch	86 (15)	59	41	7.44	0.006	40	60	1.14	0.285
Electronic pill dispenser	73 (13)	58	42	8.06	0.005	45	55	4.25	0.039
GlowCap or attachment to pill bottle	22 (4)	55	45	3.25	0.071	41	59	0.42	0.518
No digital methods	255 (44)	86	14	48.46	<0.001	33	67	0.48	0.489
Perceived importance of medication adherence									
Not at all or not very important^b^	11 (2)	64	36	28.45	0.274	36	64	35.40	<0.001
Neutral	52 (9)	83	17			62	38		
Important	197 (34)	72	28			43	57		
Very important	320 (55)	70	30			25	75		
Receptivity to storage guidance^c^									
Receptive to guidance from at least one listed source^e^	545 (94)	71	29	0.14	0.712	35	65	0.15	0.695
From physician	317 (55)	73	27	0.35	0.556	34	66	0.003	0.957
From pharmacist	383 (66)	73	27	1.34	0.247	31	69	4.96	0.026
From mobile application or website	111 (19)	79	21	4.03	0.045	32	68	0.53	0.467
From printed material (brochures)	130 (22)	88	12	23.54	<0.001	34	66	0.03	0.862
Not receptive to guidance from any listed source^f^	35 (6)	74	26	0.14	0.712	31	69	0.15	0.695

^a^ Defined as forgetting to take a medication in the last two weeks.

^b^ Values combined due to small sample size or similarity.

^c^ Non-mutually exclusive; single respondent may fall into several categories, except for composite values.

^d^ < 1% identified with an unlisted sex.

^e^ Composite variable or value.

^f^ Derived from absence of value selection.

* Pearson chi^2^; *P*-value denotes likelihood of observed chi^2^ assuming homogeneity of proportions. For non-mutually-exclusive items, chi^2^ is calculated for each selection as a binary value by the binary adherence proxy.

#### Digital medication reminders

We derived our measures indicating use of digital and non-digital medication reminder methods from two survey items from which respondents could select all that applied: non-digital methods (“Written notes,” “Post-it notes,” “Calendar,” “Chart,” “Pillbox”) and digital methods (“Smartphone app,” “Smartphone alarm,” “Siri,” “Alexa,” “Smartwatch,” “Electronic pill dispenser,” “GlowCap or attachment to pill bottle”).

#### Perceived importance of medication adherence

We measured perceived importance of medication adherence on a five-point Likert scale ranging from “not at all important” to “very important” in response to a survey item asking, “How important is it for you to take your medication as prescribed?”.

#### Medication adherent behavior

We derived our variables assessing medication adherence from questions which used memory of intaking a medication as a proxy for adherence (36). Our variables were dichotomous (“yes”, “no”) in response to survey items asking, “Have you ever forgotten to take a medication?” and “In the past two weeks, have you forgotten to take a medication?”.

#### Medication storage location

We derived our variable for storage locations of medications taken regularly from a survey item (“Where in your home do you store your prescriptions that you take on a regular basis?”) to which respondents could select all that applied: “Kitchen table,” “Kitchen cabinet,” “Kitchen counter,” “Kitchen drawer,” “In the refrigerator,” “On the bathroom vanity,” “In the vanity drawer or cabinet,” “Bathroom medicine cabinet,” “On top of the bedroom nightstand,” “In the nightstand drawer,” “Desk,” “Dining room table,” “Backpack, purse, or bag,” “Closet,” and an open-text selection for unlisted locations. We categorized open-text responses indicating use of already existing values as those values accordingly *via* consensus coding to promote interrater reliability. Table 2 displays the storage selection distribution for our sample.

**Table 2:** Home medication storage location by self-reported adherence (*n*= 580).

	Overall N (%)	Ever forgot to take a medication (%)				Recently forgot to take a medication** (%)			
Home medication storage location		Yes	No	*chi^2^* *	*P*	Yes	No	*chi^2^* *	*P*
Overall	580 (100)	72	28			35	65		
Kitchen table	58 (10)	67	33	0.59	0.443	48	52	5.43	0.02
Kitchen cabinet	146 (25)	78	22	4.09	0.043	44	56	7.55	0.006
Kitchen counter	90 (16)	69	31	0.37	0.542	38	62	0.51	0.474
Kitchen drawer	80 (14)	59	41	7.47	0.006	40	60	1.25	0.26
In refrigerator	82 (14)	61	39	5.25	0.022	38	62	0.47	0.495
On bathroom vanity	80 (14)	90	10	15.52	<0.001	36	64	0.13	0.72
In vanity drawer or cabinet	81 (14)	67	33	1.10	0.293	31	69	0.55	0.46
Bathroom medicine cabinet	112 (19)	71	29	0.07	0.791	33	67	0.13	0.72
Atop bedroom nightstand	135 (23)	61	39	10.1	0.001	38	62	0.85	0.358
In the nightstand drawer	158 (27)	56	44	24.72	<0.001	25	75	8.08	0.004
Desk	80 (14)	50	50	21.18	<0.001	33	67	0.16	0.688
Dining room table	46 (8)	61	39	2.8	0.094	37	63	0.14	0.713
Backpack, purse, or bag	64 (11)	58	42	6.67	0.01	42	58	1.89	0.169
Closet	24 (4)	79	21	0.71	0.398	29	71	0.31	0.576
Unlisted location (alone)***	14 (2)	100	0	5.7	0.017	36	64	0.01	0.922

* Pearson chi^2^; *P*-value denotes likelihood of observed chi^2^ assuming homogeneity of proportions. For all values, chi^2^ is calculated for each selection as a binary value (only affirmative displayed) by the binary adherence proxy.

** Defined as forgetting to take a medication in the last two weeks.

*** Inclusive of uncategorizable responses for which a categorizable response was not also provided by the respondent.

#### Receptivity to storage location guidance

We measured receptivity to storage guidance using a survey item (“If you received a new prescription, would you be open to receiving guidance on where to store the medication?”) to which respondents could select all that applied (“Yes, from my physician,” “Yes, from my pharmacist,” “Yes, on an app or website,” “Yes, in a brochure,” “No”). We consolidated all “yes” responses into a binary variable indicating guidance receptivity to one or more listed sources (physician, pharmacist, mobile app or website, or brochure). We cleaned “No” responses by excluding any response for whom a “Yes” response was also given for the item.

### Analysis

We assessed relative frequencies for all sample characteristics and variables listed in our *Measures* subsection. We conducted bivariate analyses for digital medication reminder method variables and variables indicating adherent behavior to medications taken regularly, receptivity to medication storage guidance, and medication storage locations currently in use by respondents. Analyses included chi^2^ tests of homogeneity of proportions and bivariate logistic regression models.

## Results

### Sample characteristics

Respondents in our middle-aged and older adult sample were 59% female and 40% male. Less than 1% of respondents identified with a non-listed sex. Respondents ranged from 40 to over 90 years old with most (64%) between the ages of 40 and 59 years. Most respondents self-identified as white (76%). Of white respondents (*n* = 439), the vast majority (95%) selected no other race or ethnicity. Just over 12% of the sample identified as American Indian or Alaskan Native, 7% identified as Black or African American, 5% identified as Asian, 5% identified as Hispanic or Latino, 2% identified as Native Hawaiian or Pacific Islander, and less than 1% identified with an unlisted race or ethnicity. Respondents’ education levels varied, though most respondents (60%) identified as having obtained a bachelor's, master's, or professional degree (Table 1).

### Digital medication reminders

Over half of respondents (56%) indicated that they use digital methods to remember to take their medication. The most common digital methods reported included use of smartphone applications or alarms (39%). The least common digital method reported was the use of pill bottle attachments, such as GlowCaps (4%).

### Perceived importance of medication adherence and adherent behavior

Most respondents (89%) indicated that they felt taking medication as prescribed was “important” or “very important,” and 65% had not forgotten to take a medication in the two weeks prior to survey. Over 71%, however, indicated that they had forgotten to take a medication at least once in their lives. Of those who had ever forgotten to take a medication (*n* = 415), almost half (48%) had forgotten to take a medication in the two weeks prior to responding to the survey.

### Home medication storage locations

The most popular home medication storage locations for medications taken regularly by our sample included nightstand drawers (27%), kitchen cabinets (25%), and atop bedroom nightstands (23%). Despite the name, only 19% of our sample stored medication in medicine cabinets. Other locations included kitchen counters (16%), in refrigerators (14%), in vanity drawers or cabinets (14%), inside of bathroom vanities (14%), in kitchen drawers (14%), inside or on top of desks (14%), in backpacks, purses, or bags (11%), on kitchen tables (10%), on dining room tables (8%), and in closets (4%). Less than 3% of respondents stored medications principally in unlisted locations (Table 2).

### Receptivity to storage guidance

Most respondents (94%) indicated that they would be receptive to guidance about where to store their medications, with most preferring guidance from pharmacists (66%) followed by guidance from physicians (55%). Fewer (19%) indicated that they would be receptive to guidance delivered on a digital platform, such as websites or mobile applications.

### Bivariate analyses

#### Medication storage locations and medication adherence

Several home medication storage locations were significantly associated with decreased odds of having ever forgotten to take a medication, including kitchen drawers (OR: 0.51, 95% CI: 0.31–0.83), in refrigerators (OR: 0.57, 95% CI: 0.35–0.93), atop bedroom nightstands (OR: 0.52, 95% CI: 0.35–0.78), in nightstand drawers (OR: 0.38, 95% CI: 0.26–0.56), desks (OR: 0.33, 95% CI: 0.21–0.54), and backpacks, purses, or bags (OR: 0.5, 95% CI: 0.29–0.85). Two home medication storage locations were significantly associated with increased odds of having ever forgotten to take a medication: kitchen cabinets (OR: 1.57, 95% CI: 1–2.45) and bathroom vanities (OR: 4.12, 95% CI: 1.94–8.76). All remaining home medication storage locations were not associated with having ever forgotten to take a medication.

Far fewer home medication storage locations were significantly associated with having forgotten to take a medication in the two weeks prior to survey. Only one location was significantly associated with decreased odds of forgetting to take a medication in the two weeks prior to survey: nightstand drawers (OR: 0.56, 95% CI: 0.37–0.84). Two locations were significantly associated with increased odds of forgetting to take a medication in the two weeks prior to survey: kitchen tables (OR: 1.9, 95% CI: 1.09–3.28) and kitchen cabinets (OR: 1.71, 95% CI: 1.16–2.51) (Table 3).

**Table 3:** Bivariate associations between home medication storage location and self-reported medication adherence.

Home Medication Storage location	Ever forgot to take a medication	Recently forgot to take a medication**
	OR (95% CI)	OR (95% CI)
Kitchen table	0.8 (0.45–1.42)	1.9 (1.1–3.28)*
Kitchen cabinet	1.57 (1.01–2.45)*	1.71 (1.16–2.51)*
Kitchen counter	0.86 (0.53–1.4)	1.19 (0.74–1.89)
Kitchen drawer	0.51 (0.31–0.83)*	1.32 (0.81–2.14)
In refrigerator	0.57 (0.35–0.93)*	1.18 (0.73–1.92)
On bathroom vanity	4.12 (1.94–8.76)*	1.09 (0.67–1.79)
In vanity drawer or cabinet	0.76 (0.46–1.26)	0.83 (0.5–1.37)
Bathroom medicine cabinet	0.94 (0.6–1.48)	0.92 (0.6–1.43)
Atop bedroom nightstand	0.52 (0.35–0.78)*	1.21 (0.81–1.8)
In the nightstand drawer	0.38 (0.26–0.56)*	0.56 (0.37- 0.84)*
Desk	0.33 (0.21- 0.54)*	0.9 (0.55–1.49)
Dining room table	0.59 (0.32–1.1)	1.12 (0.6–2.1)
Backpack, purse, or bag	0.5 (0.29–0.85)*	1.45 (0.85–2.45)
Closet	1.53 (0.56–4.18)	0.77 (0.32–1.9)

* *P *< 0.05.

** Defined as forgetting to take a medication in the last two weeks.

#### Use of digital reminder methods and medication adherence

Bivariate analyses revealed statistically significant associations between use of any digital medication reminder methods (composite variable) and having ever forgotten to take a medication (*p* < 0.001, chi^2^). Affirmative indication of having used any digital method was significantly associated with decreased odds of having ever forgotten to take a medication (OR: 0.24, 95% CI: 0.16–0.36). Significant associations were not observed between use of digital methods (composite variable) and forgetting to take a medication in the two weeks prior to survey.

Digital medication reminder methods significantly associated with decreased odds of having ever forgotten to take a medication included use of smartphone applications and alarms (OR: 0.49, 95% CI: 0.34–0.71), Apple Siri (OR: 0.26, 95% CI: 0.16–0.42), smartwatches (unadjusted OR: 0.52, 95% CI: 0.32–0.84), and electronic pill dispensers (OR: 0.49, 95% CI: 0.29–0.81). Use of other listed digital methods (Amazon Alexa and electronic attachments to pill bottles) were not significantly associated decreased odds of with having ever forgotten to take a medication.

Interestingly, while no digital reminder methods were significantly associated with decreased odds of having forgotten to take a medication in the two weeks prior to survey, some digital methods were associated with increased odds, including Amazon Alexa (OR: 2, 95% CI: 1.07–3.77) and electronic pill dispensers (OR: 1.68, 95% CI: 1.02–2.76) (Table 4).

**Table 4:** Bivariate associations between Use of digital reminders and self-reported medication adherence.

Digital reminder method	Ever forgot to take a medication	Recently forgot to take a medication**
	OR (95% CI)	OR (95% CI)
Any digital reminders***	0.24 (0.16–0.36)*	1.9 (1.1–3.28)
Smartphone app or alarm	0.49 (0.34–0.71)*	1.2 (0.85–1.71)
Apple Siri	0.26 (0.16–0.42)*	1.02 (0.63–1.65)
Amazon Alexa	0.56 (0.29–1.06)	2 (1.07–3.77)*
Smartwatch	0.52 (0.32–0.84)*	1.29 (0.81–2.07)
Electronic pill dispenser	0.49 (0.29–0.81)*	1.68 (1.02–2.76)*
GlowCap or attachment to pill bottle	0.46 (0.2–1.09)	1.33 (0.56–3.17)
No digital methods	4.19 (2.75–6.38)*	0.89 (0.63–1.25)

* *P *< 0.05.

** Defined as forgetting to take a medication in the last two weeks.

*** Composite variable.

## Discussion

### Importance of and self-reported adherence

Most respondents (89%) indicated that they felt taking medication as prescribed was “important” or “very important,” indicating that they may be more likely to be receptive to medication adherence interventions. As previously stated, adherence in this study was defined as having ever or recently forgotten to take a routine medication (36). Most respondents (65%) had remembered to take all medications in the two weeks prior to survey. Over 70%, however, indicated that they had forgotten to take a medication at least once in their lives. It is crucial that we explore how these findings relate to other studies that have explored both medication adherence and nonadherence (37).

### Home medication storage locations

We found that the most commonly used storage locations for medications taken regularly by our sample were nightstand drawers (27%), kitchen cabinets (25%), and atop bedroom nightstands (23%). These exploratory results offer support to our first hypothesis that patients utilize a variety of home storage locations to store their medications. In future work, we will learn more about the reasons and motivations underlying these choices. We suspect that some reasons may be related to home conditions unexplored in this research. For example, bedside nightstands, by virtue of where they are placed in the home, may be ideal for private medication storage over locations in communal areas. Climactic requirements or safety considerations unexplored in this research may also be factors.

The University of Michigan National Poll on Healthy Aging is the only previous study that considered storage location, although their study interest was grandchild safety (27). In total, 1,074 adults aged 50–80 with a grandchild aged 0–17 complete the University of Michigan National Poll, while 580 adults aged 40 and older completed our study analyzed here. In the two studies, researchers gave respondents different lists of location options to choose from and the naming was also different for some of the locations (e.g., “kitchen cabinet” compared to “cupboard or cabinet”). The locations reported from the Tufts University School of Medicine and the University of Michigan National Poll on Healthy Aging surveys differ greatly, which we attribute to the options available to respondents. Neither survey asked about the characteristics of the locations nor the determinants of location selection.

Our exploratory analyses suggest significant bivariate associations between several home medication storage locations and decreased odds of having ever forgotten to take a medication, including atop nightstands and inside nightstand drawers. Other locations, including kitchen cabinets, were associated with increased odds of having ever forgotten to take a medication. Fewer locations were associated with decreased odds of having forgotten to take a medication in the two weeks prior to survey. These findings offer support to our second hypothesis speculating that some storage locations are associated with greater adherence and some locations with worse adherence.

Since these unadjusted, exploratory analyses suggest that some home medication storage locations may be associated with adherence, the next step in our research is to learn what factors influence location selection, understand the implications for adherent behavior, and apply more sophisticated multivariable approaches to our analysis.

Most survey respondents (94%) indicated that they would be receptive to guidance regarding where to store their medications, with more preferring guidance from a pharmacist (66%) followed by guidance from physicians (55%). In subsequent research, we hope to learn either if the characteristics of the locations or the determinants of location selection are associated with adherence. Knowing respondents’ receptivity to guidance and knowing more about the selection of locations associated with adherence, in our next study we plan to design interventions to guide storage selection.

### Medication reminders

Several devices and apps offer medication intake reminders; however, in prior research, these devices have not been effective (33, 37, 38). There may be many reasons for this including the lack of incorporation of location as well as the focus on time-based notifications.

No known medication reminder or dispensing devices offer recommendations for optimal device storage locations, yet, since many devices use auditory and/or visual cues, we suspect that location may be a consideration for cues to be heard or seen. Further, no known devices are designed for specific locations in the home. Over half of respondents (56%) indicated that they use digital methods to remember to take their medication, the most common being the use of smartphone applications or alarms (39%). The sheer number of respondents using reminders is indicative of a perceived need, yet the lack of effectiveness in studies is concerning.

Our analysis found no significant associations between use of digital methods and forgetting to take a medication in the two weeks prior to survey. However, digital medication reminder methods that were significantly associated with decreased odds of having ever forgotten to take a medication included use of smartphone applications and alarms, Apple Siri, smartwatches, and electronic pill dispensers. Interestingly, while no digital reminder methods were significantly associated with decreased odds of having forgotten to take a medication in the two weeks prior to survey, some digital methods were associated with increased odds, including Amazon Alexa and electronic pill dispensers.

### Future directions

In subsequent analyses, we will examine other survey responses to better understand other aspects of home medication management. One question asked about the frequency with which respondents check for unused or expired medications, which may be a proxy for conscientious medication management; a reduction of clutter in a home storage location may increase adherence with fewer prescription bottles to choose from. Another question asked about the impact of the Coronavirus (COVID-19) pandemic to learn if more time at home eased medication management and thus increased adherence; alternatively, other changes, such as decreased time with pharmacists, may have reduced adherence.

Finally, in planned qualitative studies we hope to better understand how the selection of medication storage locations relate to daily routines and cues to engage in those routines (13). Through these interviews, we hope to understand the relationship between medication adherence and factors we have not previously explored, two being how adherence changes with travel and the role of patient activation, which comprises the degree of knowledge, confidence, and skills that patients have to manage their overall health (39).

## Limitations

As our survey recruitment was primarily achieved *via* an electronic mailing list compiled from Osher Lifelong Learning Institute at Tufts, our sample is not representative of the US population. This resulted in our sample appearing to be more educated and more digitally literate than the US census (40). Few studies explore the relationship directly between digital literacy and medication adherence however increased digital literacy has been correlated with increased health literacy which has been correlated to medication adherence.

As our analyses utilized self-reported adherence as measured *via* a digital survey rather than an actual measurement of adherence directly *via* observation or prescription records, response bias may have affected our results. Self-reported medication adherence has been shown to overestimate adherence behavior compared with other assessment methods and generally have high specificity but low sensitivity (41).

Additionally, our exploratory analyses were bivariate and thus did not adjust for other additional variables.

## Conclusions

Our long-term goal is to aid middle-aged and older adults in making informed choices about where to store their medications in their homes to increase adherence. Strong evidence to guide optimization of storage locations could play a crucial role in improving adherence and, thereby, the health and safety of middle-aged and older adults living independently. Our future research will investigate the relationship between medication storage locations and adherence by more fully understanding the characteristics of storage locations, the determinants of location selection, and their role in routines. With an understanding of which factors relate to storage location and impact adherence, we hope to develop best practice guidelines that can be used by pharmacists, by physician, and in innovative digital health solutions to counsel patients on optimal selection of home medication storage locations to improve their medication adherence. Our future research will also investigate how new device designs can incorporate contextual cues related to location to promote adherence more effectively. With a rising number of middle-aged and older adults and a commensurate increase in the number of patients taking prescription medications, interventions to increase adherence will lead to greater health and longevity.

## Data Availability

Raw data that support the findings of this study are available from the corresponding author, upon reasonable request.
